# Multiplex ligation-dependent probe amplification for genetic screening in autism spectrum disorders: Efficient identification of known microduplications and identification of a novel microduplication in *ASMT*

**DOI:** 10.1186/1755-8794-1-50

**Published:** 2008-10-16

**Authors:** Guiqing Cai, Lisa Edelmann, Juliet E Goldsmith, Ninette Cohen, Alisa Nakamine, Jennifer G Reichert, Ellen J Hoffman, Danielle M Zurawiecki, Jeremy M Silverman, Eric Hollander, Latha Soorya, Evdokia Anagnostou, Catalina Betancur, Joseph D Buxbaum

**Affiliations:** 1 Laboratory of Molecular Neuropsychiatry, Mount Sinai School of Medicine, New York, NY 10029, USA; 2 Seaver Autism Research Center, Mount Sinai School of Medicine, New York, NY 10029, USA; 3 Departments of Psychiatry, Mount Sinai School of Medicine, New York, NY 10029, USA; 4 Deparment of Genetics and Genomic Sciences, Mount Sinai School of Medicine, New York, NY 10029, USA; 5 INSERM U513, Paris, France; 6 Université Pierre et Marie Curie, Paris, France; 7 Department of Neuroscience, Mount Sinai School of Medicine, New York, NY 10029, USA; 8 University of Toronto, Bloorview Kids Rehab, 150 Kilgour Road, Toronto, ON, M4G1R8, Canada

## Abstract

**Background:**

It has previously been shown that specific microdeletions and microduplications, many of which also associated with cognitive impairment (CI), can present with autism spectrum disorders (ASDs). Multiplex ligation-dependent probe amplification (MLPA) represents an efficient method to screen for such recurrent microdeletions and microduplications.

**Methods:**

In the current study, a total of 279 unrelated subjects ascertained for ASDs were screened for genomic disorders associated with CI using MLPA. Fluorescence in situ hybridization (FISH), quantitative polymerase chain reaction (Q-PCR) and/or direct DNA sequencing were used to validate potential microdeletions and microduplications. Methylation-sensitive MLPA was used to characterize individuals with duplications in the Prader-Willi/Angelman (PWA) region.

**Results:**

MLPA showed two subjects with typical ASD-associated interstitial duplications of the 15q11-q13 PWA region of maternal origin. Two additional subjects showed smaller, *de novo *duplications of the PWA region that had not been previously characterized. Genes in these two novel duplications include *GABRB3 *and *ATP10A *in one case, and *MKRN3*, *MAGEL2 *and *NDN *in the other. In addition, two subjects showed duplications of the 22q11/DiGeorge syndrome region. One individual was found to carry a 12 kb deletion in one copy of the *ASPA *gene on 17p13, which when mutated in both alleles leads to Canavan disease. Two subjects showed partial duplication of the *TM4SF2 *gene on Xp11.4, previously implicated in X-linked non-specific mental retardation, but in our subsequent analyses such variants were also found in controls. A partial duplication in the *ASMT *gene, located in the pseudoautosomal region 1 (PAR1) of the sex chromosomes and previously suggested to be involved in ASD susceptibility, was observed in 6–7% of the cases but in only 2% of controls (P = 0.003).

**Conclusion:**

MLPA proves to be an efficient method to screen for chromosomal abnormalities. We identified duplications in 15q11-q13 and in 22q11, including new *de novo *small duplications, as likely contributing to ASD in the current sample by increasing liability and/or exacerbating symptoms. Our data indicate that duplications in *TM4SF2* are not associated with the phenotype given their presence in controls. The results in PAR1/PAR2 are the first large-scale studies of gene dosage in these regions, and the findings at the *ASMT *locus indicate that further studies of the duplication of the *ASMT *gene are needed in order to gain insight into its potential involvement in ASD. Our studies also identify some limitations of MLPA, where single base changes in probe binding sequences alter results. In summary, our studies indicate that MLPA, with a focus on accepted medical genetic conditions, may be an inexpensive method for detection of microdeletions and microduplications in ASD patients for purposes of genetic counselling if MLPA-identified deletions are validated by additional methods.

## Background

Autism spectrum disorders (ASDs) are pervasive developmental disorders that include autism, Asperger syndrome, and pervasive developmental disorder-not otherwise specified (PDD-NOS). ASDs are characterized by varying degrees of impairment in communication and social interactions, as well as by the presence of restricted, repetitive and/or stereotyped patterns of behavior, interests, and/or activities, with signs typically manifesting by the age of 3. Recent epidemiological studies report a prevalence of ASD of 3–6 per 1000 children [1,2]. Males have a higher risk than females, with a male to female ratio of ~4:1. Twin studies show a concordance rate of 60%–90% for monozygotic twins and 0–10% for dizygotic twins, and the genetic liability for autism is estimated to be more than 90% [3]. In spite of the high heritability, the identification of genetic factors in ASDs has proved difficult, due at least in part to the fact that ASDs are characterized by a high degree of genetic heterogeneity.

In the past, approximately 10% of individuals with ASDs could be diagnosed as having secondary autism, in which a chromosome abnormality, single-gene disorder, or known environmental agent was identified as causative [3,4]. However, more recent studies suggest that chromosome abnormalities and single-gene disorders are more common as causes of ASDs [5,6]. Many children with ASD have some degree of cognitive impairment (CI) and genetic disorders associated with CI have also been associated with ASDs. Thus, ASDs have been reported as possible manifestations of genetic syndromes resulting in CI, including fragile X syndrome, tuberous sclerosis, Angelman syndrome, Prader-Willi syndrome, 22q11 deletion/DiGeorge syndrome, Cohen syndrome, Smith-Lemli-Opitz syndrome, Smith-Magenis syndrome, Williams syndrome, Sotos syndrome, and Klinefelter syndrome [7-10].

Chromosomal abnormalities throughout the genome have been reported in cases of ASD [11]. In addition to traditional methods like FISH to detect chromosome abnormalities, new array-based techniques allow robust whole genome scanning for copy number variants (CNVs) and have resulted in the identification of new disease genes [12-14]. For example, using Affymetrix 10 K SNP arrays, the Autism Genome Project Consortium identified deletions in the neurexin 1 gene (*NRXN1*) in 2p16 as a cause for ASD [15], now described in further studies [13,16,17]. More recently several groups have reported deletions in 16p11 in ASD [13,14,18]. Multiplex ligation-dependent probe amplification (MLPA) is a recently developed method to accurately quantify levels of diverse DNA sequences based on target-directed ligation followed by quantitative polymerase chain reaction (Q-PCR), and can be used to test for the presence of chromosomal abnormalities that lead to changes in copy number. For each sequence of interest, the two probes are joined by the use of a ligase only if they are both bound to a specified target sequence. The resultant ligated product is then amplified by Q-PCR in a manner proportional to the initial levels. MLPA has been proposed as a rapid and cost-effective method for routine screen of chromosome abnormalities in mental retardation [19,20]. To determine the degree to which chromosome abnormalities associated with CI contribute to autism, and to evaluate MLPA for genetic counselling, 279 unrelated ASD subjects were screened for CI-associated deletions using MLPA.

## Methods

### Subjects

A total of 279 unrelated individuals with an autism or autism spectrum diagnosis were recruited by the Seaver Autism Research Center (SARC) and/or the Autism Genetic Resources Exchange (AGRE). All parents provided written informed consent. Affected status conformed to the following categories, used by AGRE and by other researchers: autism, not quite autism, and broad spectrum. Individuals who met Autism Diagnostic Interview-Revised (ADI-R) criteria for autism were categorized as autism [21-23]. The term 'not quite autism' refers to individuals who are no more than one point away from meeting autism criteria according to the ADI-R on any or all of the 3 content domains (social, communication, and/or behaviour) and meet the age of onset criterion (before 36 months); or individuals who meet criteria on all 3 domains, but do not meet criteria for age of onset. The term 'broad spectrum' defines individuals who show patterns of impairment along the spectrum of pervasive developmental disorders, which encompasses individuals ranging from mildly- to severely- impaired. Given this broad classification and as there was in many cases sufficient clinical information to assign a diagnosis of pervasive developmental disorder-not otherwise specified (PDD-NOS) or Asperger disorder (based on DSM-IV criteria) to some of these individuals, these classifications are also enumerated. Subjects had been previously subjected to a routine work-up to exclude medical conditions, including major chromosomal abnormalities, as well as fragile X syndrome and tuberous sclerosis. Subjects with such abnormalities were excluded from the current study.

The 279 individuals screened included 254 from multiple affected (multiplex) families, and 25 from singly affected (simplex) families. The sex distribution in the sample was 3.9 males to 1 female (222:57). Within the sample, 205 were self-identified as Caucasian, 6 African American, 24 Hispanic or Latino, 5 Asian, and 11 of mixed ethnicity, with 28 of an unknown ethnicity. There were 270 subjects with a diagnosis of autism, 2 with Asperger syndrome, 1 with PDD-NOS, 3 with NQA and 3 with broad spectrum diagnoses. Average age at last evaluation was 7.94 ± 5.79 years old. Amongst ninety subjects evaluated for IQ with the Raven, sixteen (17.8%) had IQ scores below 70 and 10 (11.1%) had scores between 70–79. When an abnormal MLPA probe signal was observed, all available family members were included in subsequent analyses. All studies were approved by the Program for the Protection of Human Subjects of Mount Sinai School of Medicine (GCO#06-0688).

### MLPA

MLPA probe sets targeting chromosomal regions previously associated with CI were purchased from MRC-Holland (Amsterdam, Netherlands) and used according to the manufacturer's protocols. Briefly, 50–200 ng genomic DNA in 5 μl water was denatured, mixed with a probe set (SALSA MLPA Kits P064-MR1, P096-MR2, P106-MRX, or P018-SHOX) and high salt MLPA buffer. The mixture was hybridized at 60°C for 16 h. The ligation reaction was then performed and 5 μl of the ligation reaction product was used for PCR. Subsequently, PCR products were subjected to capillary electrophoresis on an ABI 3130 genetic analyzer (Applied Biosystem, Foster City, CA, USA). Raw traces from the electrophoresis were imported into GeneMarker software (SoftGenetics, State College, PA, USA) for MLPA analysis. After population normalization, data were compared to two different controls: 1) a single control sample, representing the sample with the fewest abnormal calls in each experiment; and, 2) a synthetic control sample, which represents the median of all normal samples in each experiment. A threshold of dosage change <0.75 was used to identify potential deletions, and a threshold >1.30 was used to identify potential duplications. All samples with potential deletions or duplications were re-analyzed by MLPA. To analyze the 15q11-q13 interval, a methylation-specific multiplex ligation-dependent probe amplification (MS-MLPA) kit (SALSA MLPA kit ME028 PWS/AS, from MRC-Holland) was used. This kit contains 12 methylation-sensitive probes in 15q11-q13, of which 5 are subject to imprinting, as well as 3 methylation-sensitive probes in other regions.

### FISH

Commercially available locus specific probes, TUPLE1 (22q11.2), D15S11 (15q11.2), SNRPN (15q11.2), and GABRB3 (15q11.2) were purchased from Vysis (IL). BAC DNAs for validation of MLPA results were obtained directly from BAC PAC resources (CA). Each BAC was labelled using the Nick Translation Reaction Kit and Spectrum Green-11-dUTP (Vysis, IL, USA). Single coloured FISH was performed on metaphase spreads and interphase nuclei from established lymphoblastoid lines dropped onto pre-cleaned glass slides. Co-denaturation and hybridization of probes and slides was performed at 73°C for 3 minutes and 37°C overnight, respectively, using the HyBrite hybridization system (Vysis, IL, USA). After hybridization and washing, chromosomes and nuclei were counterstained with 0.5 μg/ml DAPI (4',6-Diamidino-2-Phenylindole Dihydrochloride) in Vectashield (Vector Laboratories, CA, USA) and a cover slip was applied to the slide. Images were captured with an ImagePoint cooled CCD video camera (Photometrics, AZ, USA) through a Labophot-2A florescence microscope (Nikon, NY, USA) using Cytovision FISH software (Applied Imaging, CA, USA). Generally, 3 to 5 metaphases were examined and 50 – 100 interphase nuclei were scored for the number of signals present for each probe.

### Q-PCR and Direct Sequencing

Validation of samples with apparent deletions/duplications included direct sequencing, Q-PCR and/or fluorescent in situ hybridization (FISH). Genomic Q-PCR using the Universal Probe Library (UPL) probe (Roche, IN, USA) was performed to confirm microdeletions or microduplications. Probes were selected using the ProbeFinder v2.04 software (Roche, IN, USA). Quantitative PCR was performed on an ABI Prism™ 7900 HT sequence detection system (Applied Biosystems, CA, USA). Each sample was analyzed in quadruplicate in a reaction including 25 ng genomic DNA, 200 nM of each primer, 100 nM UPL probe, and 1× Platinum Quantitative PCR SuperMix-Uracil-N-Glycosylase (UDG) with ROX (Invitrogen, CA, USA). The values were evaluated using the Sequence Detection Software v2.1 (Applied Biosystems, CA, USA). Data analysis was performed using qBase [23]. Reference genes chosen from COBL, GUSB, PPIA, SNCA were included based on the minimal coefficient of variation and then data was normalized by setting a normal control to a value of 1.

## Results

We made use of four commercial MLPA panels (MR1, MR2, MRX, and SHOX, which included 84 probes directed at autosomal loci and 46 probes directed at X-linked loci, 4 probes at Y-linked loci, and 27 probes directed at PAR1/PAR2), covering many of the chromosomal regions associated with known, recurrent deletions/duplications associated with CI [see Additional file 1].

MLPA screening identified increased or decreased signal in 28 subjects out of the 279 screened, consistent with potential microduplications or microdeletions. We observed potential duplications in 15q11-q13 (4 subjects), 22q11 (2 subjects), the *TM4SF2 *gene in Xp11 (2 subjects), and the *ASMT *gene in Xp22.32 (PAR1) (16 subjects). We observed potential deletions in the *ASPA *gene in 17p13 (1 subject), the *PAX6 *gene in 11p13 (1 subject), the *EXT1 *gene in 8q24 (1 subject), and the *ARHGEF6 *gene in Xq26 (1 subject).

### Copy number variation in 15q11.1-q13

**Figure 1:**
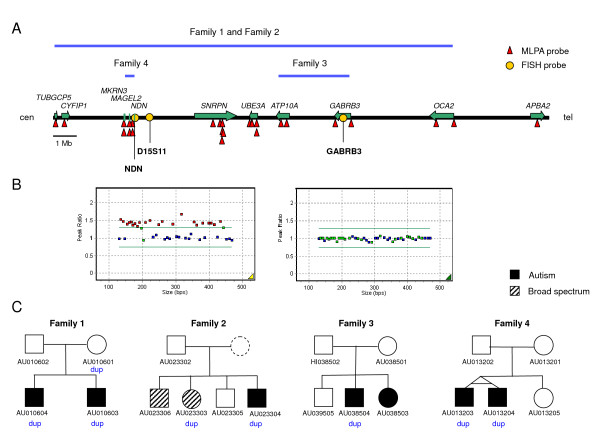
**Chromosome 15q11-q13 duplications in four families**. A. Schematic representation of the region, highlighting the extent of the duplications in the four families (blue lines), the localization of the MLPA probes (red triangles) and FISH probes (yellow ovals) used, and the genes within the interval along with their orientation (green arrows). B. MLPA peak ratio for 15q11-13 (red and green) and control (blue) MLPA test probes in Family 1 individuals AU010604 (left) and AU010602 (right). Red, probes with peak ratios > 1.3; green, probes with peak ratios <= 1.3. In the left panel, the green rectangle with a peak ratio of ~1 corresponds to *APBA2*. C. Segregation of the duplications in the four families.

The most common, autosomal, potential CNV observed by MLPA was variation in the 15q11-q13 region, where four unrelated probands demonstrated apparent duplications (Figure 1A, B, C) [clinical information is summarized in Additional file 2]. This region, previously associated with PWA and ASDs, was covered by four probes, one each in the *TUBGCP5*, *MKRN3*, *NDN *and *GABRB3 *genes. Probands 1 (AU010604) and 2 (AU023304) showed duplications in all probes, proband 3 (AU038504) had a duplication in the probe in *GABRB3*, and proband 4 (AU013203) had a duplication in the probes in *MKRN3 *and *NDN*.

Two common FISH probes, D15S11 and *GABRB3*, were used to validate the duplications. Probands 1 and 2 showed duplications with both FISH probes, proband 3 showed duplication with the *GABRB3 *FISH probe but not the D15S11 probe, and proband 4 did not show duplication with either FISH probe (data not shown). Proband 4 also showed normal hybridization with the *NDN *FISH probe RP11-494F2, suggesting that the duplication might be too small to be identified by FISH.

To further evaluate these duplications, 21 additional MLPA probes within the 15q11-q13 region were used; the additional probes were directed to *CYFIP1 *(1 probe), *MKRN3 *(1 probe), *MAGEL2 *(2 probes), *NDN *(1 probes), *SNRPN *(6 probes), *UBE3A *(4 probes), *ATP10A *(2 probes), *GABRB3 *(1 probe), *OCA2 *(2 probes), and *APBA2 *(1 probe). Probands 1 and 2 showed signal increases with all of these probes except for the probe in *APBA2 *(Figure 1B), indicating that the duplication included a 5.7 Mb region comprised between breakpoints 1 and 3 [24,25]. Proband 3 showed increases only for the probes in *ATP10A *and *GABRB3*, encompassing a 1 Mb region. Finally, proband 4 showed increases only for the probes in *MKRN3*, *MAGEL2 *and *NDN*, encompassing a limited region of 120 kb.

We next examined how these confirmed duplications segregated in the families. In the family of proband 1 (Family 1), the duplication was maternally inherited, and was also found in an affected brother. In Family 2, the proband had a brother and a sister – both with a broad spectrum diagnosis, with the sister, but not the brother, carrying the duplication. The father and an unaffected brother did not harbor the duplication, while the mother's DNA was not available. In Family 3, the duplication was not found in an affected sister or in either parent. Confirmation of paternity and maternity by genotyping microsatellite markers indicated that the duplication likely arose *de novo*. In Family 4, the duplication was found in the affected, monozygotic twin, but in neither parent. Again, confirmation of paternity and maternity indicated that the duplication likely arose *de novo*.

We subsequently used methylation-sensitive MLPA to further characterize these rearrangements. These analyses confirmed that the duplication in the two affected children in Family 1 was of maternal origin. In contrast, the origin of the duplication in the mother is paternal, consistent with an absence of phenotype in her. Methylation analysis in children in Family 2 demonstrated a maternal origin. For Family 3, neither the *ATP10A *nor *GABRB3 *probes are methylation-sensitive, so the analysis could not provide further data. Analysis in Family 4 demonstrated the *de novo *duplication of the *NDN *gene was maternally-derived.

### Copy number variation in 22q11.2

**Figure 2:**
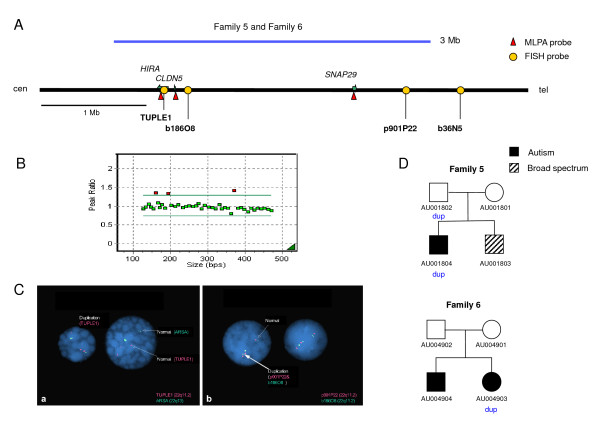
**Chromosome 22q11 duplication in two families**. A. Schematic representation of the region, highlighting the extent of the duplications in the two families (blue line), the localization of the MLPA probes (red triangles) and FISH probes (yellow ovals) used, and the genes within the interval along with their orientation (green arrows). B. MLPA peak ratio for 22q11 MLPA test probes in Family 5 individual AU001804. Red, probes with peak ratios > 1.3, corresponding to *HIRA*, *CLDN5 *and *SNAP29*; green, probes with peak ratios <= 1.3.C. FISH results of the 22q11 duplication. a, test probe *TUPLE1 *(red) in 22q11 shows three copies, whereas the control probe *ARSA *(green) in 22q13 shows two copies. b, both test probes b186O8 (green) and p901P22 (red) in 22q11 show three copies. D. Segregation of the duplication in the two families.

Two cases (probands 5-AU001804 and 6-AU004903) with an apparent duplication in 22q11.2 were observed in this study (Figure 2A, D) [see Additional file 2]. In both cases, all three probes that were screened in the region (in *HIRA, CLDN5 *and *SNAP29*) were duplicated (Figure 2B). FISH analysis was used to confirm the duplications (Figure 2C) using a commercial test probe (*TUPLE1, Vysis*), two additional test probes (b186O8 and p901P22) within the duplication, one probe (b36N5) (data not shown) to define the distal breakpoint and one control probe (*ARSA, Vysis) *in 22q13. All three test probes (*TUPLE1*, b186O8 and p901P22) were clearly triplicated, demonstrating a duplication in 22q11.2 with a direct orientation, corresponding to the longer (3 Mb) of the two recurrent CNVs observed in this region [26].

Examination of other family members indicated that in Family 5, the duplication was inherited from the father, but a brother, with a diagnosis of broad spectrum, did not have the duplication. In Family 6, the proband, a girl with a diagnosis of autism carried the duplication, but an affected brother did not. Furthermore, the duplication was not observed in either parent. Confirmation of paternity and maternity by microsatellite markers indicated that the duplication likely arose *de novo*.

### Additional copy number variation in an autosomal locus

One additional locus showed validated copy number changes in the autosomes. Seven probes were screened in the region associated with Miller-Dieker lissencephaly syndrome and Canavan disease in 17p13-pter, in *HIC1*, *MGC3329*, *PAFAH1B1*, *ASPA*, and *TRPV1*. In a boy with a diagnosis of autism, we observed an apparent deletion in a probe within the *ASPA *gene involved in Canavan disease. The apparent deletion was also observed in the boy's monozygotic twin, and was maternally inherited. Q-PCR analysis confirmed the presence of a 12.1 Kb deletion, encompassing part of intron 3 and extending through exon 5, on one allele of the *ASPA *gene. Sequencing the second allele did not identify any coding abnormalities, indicating that the boys were not compound heterozygotes for coding variants.

### Copy number variations in X-linked loci

Of the 77 probes directed to the sex chromosomes, 2, in *TM4SF2 *and in *ASMT*, showed copy number changes.

**Figure 3:**
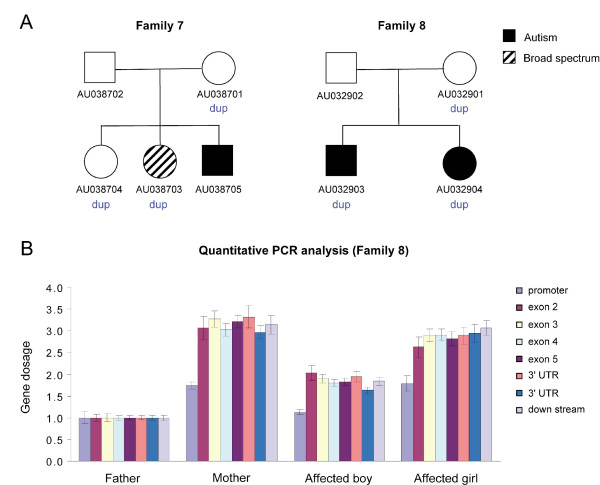
**Partial duplication of X-linked *TM4SF2 *gene in Xp11.4**. A, Segregation of the duplication in the two families with duplication of the *TM4SF2 *gene. B. Genomic quantitative PCR of Family 8 using eight probes across the *TM4SF2 *gene; similar results were obtained in Family 7 (not shown).

A girl with a diagnosis of broad spectrum (proband 7-AU038703) and an unrelated boy (proband 8-AU032904) with a diagnosis of autism were found to have an apparent duplication for a single probe (out of 2) in the X-linked gene *TM4SF2 *(Figure 3). The probe mapped to exon 5 of the gene, while the probe showing no apparent change was 114 kb away, on exon 1. In the case of the girl, the duplication was maternally derived, and was also found in an unaffected sister, but not in an affected brother, while in the case of the boy the apparent duplication was found in an affected sister, and was also maternally derived. Genomic Q-PCR using the Universal Probe Library (UPL) probe was performed to further characterize the duplication. The *TM4SF2 *gene is comprised of 8 exons, with exon 1 — containing the ATG — followed by a 104 kb intron, with the remaining exons and introns covering an additional 23 kb region. UPL probes of the *TM4SF2 *gene, located in the promoter, 480 bp before exon 1, one each in exons 2, 3, 4, and 5, and two in the 3' UTR (exon 8), and one probe 2422 bp down stream to the gene, were tested (a probe in exon 1 was also tested, but it failed in Q-PCR). All probes showed increased dosage change, except the probe in the promoter, in agreement with the MLPA results.

As it remained uncertain as to whether a duplication in *TM4SF2 *represented a risk factor for CI (see Discussion), we carried out genomic Q-PCR in 248 (174 males and 74 female) Caucasian controls (both ASD families carrying the *TM4SF2 *duplication were of Caucasian ancestry). We identified the duplication in 2 male and 4 female controls, making it very unlikely that the duplication plays a pathogenic role in CI and/or ASD.

### Copy number variations in PAR1

**Figure 4:**
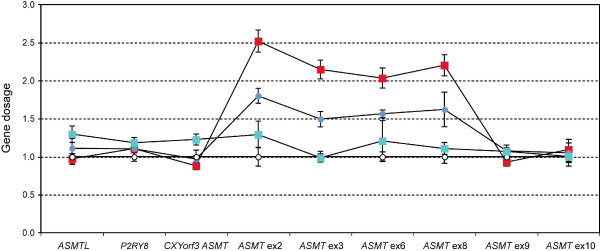
**Quantitative PCR mapping for the *ASMT *gene duplication**. Quantitative PCR probes were designed to detect copy number changes in *ASMTL*, *P2RY8*, *CXYorf3 *(which overlaps with exon 1 of *ASMT*), and *ASMT *(exons 1, 2, 3, 6, 8, 9, and 10). Results for a normal control (white), a subject with ASD but without the duplication (green), a subject with ASD with the duplication (i.e., three copies) (blue), and a subject with ASD with four copies of the region (red) are shown.

Sixteen unrelated ASD subjects (5.4%) were found to have an apparent duplication for the probe in exon 8 of the *ASMT *gene, which localizes at the end of the PAR1 region. All other probes in the PAR1 region (targeting the *SHOX*, *CSF2RA *and *IL3RA *genes) showed normal gene dosage. To both validate and map the duplication, Q-PCR probes and assays were designed to across the interval, targeting both neighbouring or overlapping genes (*ASMTL, P2RY8*, and *CXYorf3) *as well as additional *ASMT *exons. Q-PCR revealed that the duplication includes a ~18 kb fragment spanning exon 2 to exon 8 of the *ASMT *gene (Figure 4). To ensure that the observed duplication was not due to copy number artefacts arising in transformed lymphoblastoid cell lines, blood DNA from an additional 30 unrelated subjects with ASD were further screened, and the duplication was found in 2 such cases (6.7%).

Screening of 248 Caucasian controls identified 4 such duplications (1.6%), reflecting a significant increase in ASD as compared to controls. Comparing the Caucasian cases in the first screen (n = 222) with the Caucasian controls (n = 248) demonstrated significant differences (Fisher Exact test P = 0.003). Furthermore, four subjects with ASD showed four copies of the duplicated region, which were not observed in any controls. In every case where it could be determined in the ASD families, segregation analysis showed that the duplication was transmitted (9 cases from the mother, 7 from the father, with the remaining samples missing at least one parent). ADI-R scores were available in thirteen families [see Additional file 3].

### Apparent copy number variations due to altered probe binding

In three cases, apparent deletions identified by MLPA were subsequently shown to be single-base changes that altered the binding of the MLPA probes. Eight probes were screened in the 11p13 region associated with WAGR syndrome, in *BDNF *(2 probes), *PAX6 *(3 probes), *WT1 *(2 probes) and *HIPK3 *(1 probe). An apparent deletion of a single probe in *PAX6 *was observed in an affected boy, and in his affected brother and sister (all with a diagnosis of autism). The apparent deletion was paternally derived. Sequencing of the region to which the MLPA probes bound demonstrated a synonymous single base change (c.545 C→T) in exon 5 in all individuals with the apparent deletion, indicating that reduced binding of the probe was the cause for the apparent deletion. This rare variant hasn't been previously described.

Six probes were screened in the 8q24 region associated with Langer-Giedion syndrome, in *TRPS1 *(2 probes), *EIF3S3 *(1 probe), *EXT1 *(2 probes) and *SAMD12 *(1 probe). An apparent deletion of a single probe in *EXT1 *gene was observed in an affected boy, in one of his brothers with autism, and in two of three unaffected siblings. Neither parent showed the apparent deletion; however, microsatellite analysis confirmed maternity but not paternity. Sequencing the probe binding region identified a c.3089 C→G change in 3' UTR of the *EXT1 *gene, which segregated with the altered binding of the MLPA probe and hasn't been previously described.

An apparent deletion for a probe in exon 9 of *ARHGEF6 *on Xq26 was found in a boy with autism. MLPA probes in exon 1, exon 4 and exon 18 showed normal dosage. The apparent deletion was maternally derived, and was also found in an affected brother. Sequencing of exon 9 identified a synonymous single base change (c.1404 C→T) not described previously in the region of probe binding that segregated with the altered MLPA results.

## Discussion

In this study, 279 unrelated subjects with ASDs were investigated for microdeletions and microduplications associated with CI using MLPA. The cohort was ascertained for genetic studies with exclusion criteria including a prior medical diagnosis. Two hundred and fifty-four of the subjects screened were from multiplex families. We made use of four commercial MLPA probe kits, which captured many of the microdeletions and microduplications previously associated with CI and, in some cases, with ASDs. A focus on known microdeletions and microduplications makes the results most relevant to genetic counselling. In general, our study found ~1–2% of the cases had a chromosomal abnormality previously associated with CI. This yield suggests that directed MLPA, expanded to include additional ASD loci, would represent one option for clinical testing as the cost is substantially smaller than genome-wide arrays. Of course, additional methods will be required for the detection of point mutations in the relevant genes as MLPA will not readily detect point mutations.

We identified 4 duplications in chromosome 15, two typical and two atypical. Chromosome 15q11-q13 duplications are the most common alteration in autism in prior studies [11,27-30]. The most frequently reported pattern of 15q11-q13 duplications in autism is supernumerary marker chromosome 15 or isodicentric 15 [idic(15)], with two or more extra copies of this region including the PWA critical region and variable in size [28,29]. In this study, we did not find any individuals with this type of duplication, probably because these abnormalities are identified by karyotyping, and such individuals would have been excluded in the cohort. All four duplications we found were interstitial microduplications. The extent of these four duplications was confirmed by dense MLPA probes. Two of these duplications corresponded to the well-defined duplication covering the PWA critical region between breakpoints 1 and 3 and have been reported in many individuals with autism or ASD [24,25]. This region is subject to imprinting and maternal duplications are associated with ASD, while paternal duplications usually have a normal phenotype [30]. The two novel 15q11-q13 duplications were situated on each side of PWA critical region, on 15q11.2 and 15q12, respectively.

One of the novel duplications (proband 3) is ~1 Mb in size adjacent to the *UBE3A *gene, which is the gene responsible for Angelman syndrome. The duplication encompasses two potential autism susceptibility genes, *GABRB3 *and *ATP10A*. Several association studies have suggested that *GABRB3 *could be an autism susceptibility gene [31-33]. For example, we and others previously reported that there is a significant association between a polymorphism of the *GABRB3 *gene and ASD (and see discussion therein) [33]. Another group reported *GABRB3 *associated with ASD and found evidence for linkage in a subgroup of patients with an elevated score on the "insistence on sameness" factor [34]. A recent functional study using ASD brain samples demonstrated that *GABRB3 *is subject to epigenetic dysregulation: the product of the *MECP2 *gene, which when mutated can cause Rett syndrome, binds to methylated CpG sites within *GABRB3 *and acts as a chromatin organizer for optimal expression of both alleles of *GABRB3 *in neurons [35]. *ATP10A *is a P-type ATPase gene maternally expressed. A linkage disequilibrium study in the 15q11-q13 region found preferential transmission of a haplotype of *ATP10A *to ASD subjects [36]. The *de novo *origin of this duplication in patient 3 is consistent with a potentially causal role in ASD.

Another novel small duplication (proband 4) was located adjacent to *SNRPN*, a gene implicated in Prader-Willi syndrome (PWS). Three small genes, *MKRN3*, *MAGEL2 *and *NDN*, are situated within this very circumscribed region. *MKRN3 *codes a ring finger protein while *NDN *and *MAGEL2 *belong to the same *NDN*/*MAGE *gene family. All three genes are expressed exclusively from the paternal allele and are thought to be implicated in some clinical features of PWS [37-39]. *MAGEL2 *is important in regulating the circadian rhythmicity related to some features of PWS [40,41]. *NDN *also plays a role in facilitating the differentiation and specification of GABAergic neurons in cooperation with Dlx homeodomain proteins [42]. Again, the *de novo *origin of the duplication in family 4 is consistent with a potentially causal role in ASD.

In a recent review that diagrammed all the cytogenetic abnormalities reported in cases of autism [11], many cases of 15q11-q13 duplications with variable size overlapped our two novel duplications. Candidate genes in these two causal regions may implicate aberrant glutamate signalling interacting with epigenetic factors in some ASD cases. However, segregation studies complicate simple explanations of causality. In Family 3, the small duplication of *ATP10A *and *GABRB3 *was found in one affected male proband but not in his affected sister, whereas in Family 4 the duplication of *MKRN3*, *MAGEL2 *and *NDN *was found in the affected monozygotic twins. However, it is again important to note that both of the novel duplications arose *de novo*, providing support for a causal or contributory role for these genomic variants to ASD. However, the mechanism by which these novel duplications may contribute to ASD needs further analyses.

Two subjects with a 22q11.2 duplication cognate to the 22q11 deletion syndrome or DiGeorge syndrome were observed. Several studies reported a high rate (20%–50%) of autistic spectrum disorders in children with 22q11.2 deletion syndrome [43-45]. Recently, the 22q11.2 duplication was found as a highly variable syndrome [26,46]. The majority of patients with 22q11 duplications have cognitive deficits including speech delay and developmental delay, although the penetrance is variable [47]. The 22q11 duplication was also associated with variable presentation in our study. The duplication was found in a male proband with autism but not in an affected brother with broad-spectrum disorder in one case, while it was present in a female proband with autism but not in her autistic brother in the second case. In Family 5, the reportedly healthy father was found as a carrier of duplication. Phenotypically normal parents of 22q11.2 duplication children also were reported in other studies as carriers of this duplication [26]. Notably, in Family 5, the duplication was associated with a more severe phenotype (narrow autism versus board spectrum disorder), while in Family 6, a girl (perhaps with a higher threshold for liability) carried the duplication but her autistic brother did not. It is widely recognized that deletion of 22q11 region is related to CI and psychiatric symptoms, including delayed motor and speech-language development, mental retardation, impaired spatial reasoning, attention-deficit hyperactivity disorder, autism spectrum disorders, mood disorders, and/or schizophrenia spectrum disorders [48,49]. Multiple genes within this causal region have been identified playing a role in neuronal cognitive development [50-52], with TBX1 implicated in ASD [52]. Thus, the 22q11.2 duplication might also function as a risk factor for ASD and increase the liability or severity in ASD individuals.

ASDs can share characteristics with Williams-Beuren syndrome and a recent study of 128 children with WBS identified nine with ASD [53,54]. Duplications of the WBS critical region in 7q11.23 have also been described in several patients with ASD, severe language delay and mental retardation [55]. Another study reported a case with a diagnosis of ASD and an atypical deletion of WBS interval [56]. Similarly, a 17p11 deletion can cause Smith-Magenis syndrome, and 17p11 duplication is now recognized as a new syndrome of mental retardation, often associated with autistic features [57,58]. We did not observe any deletion/duplication in these regions in our sample, suggesting that these gene dosage anomalies are rare contributors to ASDs.

One subject and his monozygotic twin were found to be partially deleted for one allele of aspartoacylase (*ASPA*) gene, while the second allele was intact. Mutations of *ASPA *gene cause Canavan disease [MIM608034], an autosomal recessive disorder. Both subjects in our study have a diagnosis of autism, and their mother, determined as unaffected, also carries this deletion. We assume that this deletion is not associated with the ASD phenotype.

Fragile X syndrome is the most common syndrome related to ASD. The prevalence of autism or autistic features in individuals with fragile syndrome is about 25–33%, and the prevalence of fragile X in autism is estimated at 2% [8,9]. Fragile X is a single-gene disorder typically caused by inactivation of the *FMR1 *gene at Xq28 typically by trinucleotide repeat expansion. In our study, no gene dosage abnormality was found in *FMR1 *and *FMR2*, but note that expansions would not be detected by MLPA.

In addition to the FMR genes, other genes related to X-linked mental retardation were screened in this study. *TM4SF2 *or tetraspanin 7 (*TSPAN7*) at Xp11.4 was found partially duplicated in two unrelated subjects with autism in our sample. Q-PCR confirmed that the duplication included exons 2 to the down stream of *TM4SF2 *gene, but not exon 1. Probes of *PQBP1 *gene on Xp11.23, and probes of *ARX*, *IL1RAPL1 *and *RPS6KA3 *genes on Xp22 showed normal dosage. This indicates that a microduplication exists in Xp11 with a breakpoint within the *TM4SF2 *gene. The protein encoded by the *TM4SF2 *gene mediates signal transduction events that play a role in the regulation of cell development, activation, growth and motility [59]. It is known to form a complex with integrins [60], and two integrin genes, integrin beta 3 (*ITGB3*) and integrin beta 4 (*ITGB4*), were reported associated with ASDs [61,62]. There is some evidence for a role for *TM4SF2 *in mental retardation. The first study identified a translocation in this gene in a female patient with mild mental retardation associated with minor autistic features, as well as mutations (a premature stop codon and a missense mutation, both maternally inherited) in 2 out of 33 small families with X-linked mental retardation [60]. Note, however, that a subsequent study questioned the role of the gene and particularly one of the missense mutations (P172H) identified in the first study, because further linkage analysis based on cognitive status appeared to exclude the region of the *TM4SF2 *gene [63]. However, this same variant has subsequently been observed in an additional individual with mental retardation [64], but not in 320 controls with similar ethnic background. An additional deletion in the gene has also been identified in mental retardation [65]. A recent study reported *TM4SF2 *duplication in two patients, one with syndromic and the other with nonsyndromic mental retardation [66], but suggested that this duplication might be a neutral polymorphism as there was no evidence for skewed X-chromosome inactivation in the unaffected mothers carrying the duplication. We have now identified this same duplication in both male and female unscreened control subjects, supporting the interpretation that this duplication is a neutral polymorphism.

In individuals with Klinefelter syndrome (47, XXY), levels of ASD traits are significantly higher across all dimensions of the phenotype, which suggests that gene dosage changes on X chromosome might play a role in ASD behaviours [10,67]. Similarly, individuals with an XYY karyotype have been reported to have delayed speech and language skills or ASD [68]. The pseudoautosomal regions (PAR1 and PAR2) are homologous across the X and Y chromosomes. Deletions of these regions have been reported in three females with ASD [69]. In addition, the gene for acetyserotonin O-methyltransferase (*ASMT*), located in PAR1, has been reported to be associated with abnormal melatonin synthesis in ASD [70,71].

The PAR1 and PAR2 regions are not well-represented in some arrays used for genome-wide studies and our MLPA results represent the first extensive analysis of these regions in ASD. We found a duplication in the *ASMT *gene to be significantly associated with ASD in the current sample. Follow-up genetic studies in additional cohorts would be worthwhile to confirm our finding. Further studies on genetic variation in the *ASMT *gene, the effects of the CNV on gene expression, and the relationship of this CNV to both ASDs and sleep disorders are warranted.

Finally, our study showed that MLPA can be a useful, inexpensive tool to evaluate clinically significant chromosomal microdeletions and microduplications in ASD and associated disorders. It is likely that with a targeted panel of MLPA probes, clinical genetic diagnoses can now be made in over 10% of ASDs cases. MLPA is considerably less expensive when compared to FISH and to aCGH-based method; and it can be efficient even when compared to Q-PCR. MLPA is also an accepted approach in New York State (NYS), which has not yet approved large-scale aCGH-based tests. MLPA can also be used to validate aCGH or microarray findings.

We have identified one limitation of MLPA, in that there can be a loss of signal in the case of a single base change within a probe-binding site. In the current study, three apparent deletions were later identified to be caused by single-base sequence changes in probe binding regions, indicating that MLPA results indicating a potential deletion in a single probe should be followed-up by additional studies. Although none of these variants that we identified were observed in 49 Caucasian controls (data not shown) (nor were they found in the NCBI SNP database), the variants were non coding (two, in *PAX6 *and *ARHGEF6 *resulted synonymous amino acid changes, and one was located in the 3' UTR of *EXT1*), and are variants of unknown significance.

## Conclusion

In summary, we used MLPA as a screen and identified known and novel chromosomal abnormalities in ASD. We observed instances of microduplications in 15q11-q13, including two novel microduplications, and microduplications in 22q11.2. We also identified duplications in *ASMT*, which may contribute to ASD risk or ASD-associated traits. Compared to array based methods, MLPA, with subsequent validation of potential deletions, is a relatively practical, inexpensive and fast tool for screening for chromosome rearrangements in ASDs. It is important to validate deletions identified with MLPA by the use of multiple probes and/or additional technical approaches.

## Competing interests

The authors declare that they have no competing interests.

## Authors' contributions

GC was involved in the design and performance of the study and drafted the manuscript. LE and NC performed the FISH studies. JEG was involved in the MLPA experiments. AN was involved in the Q-PCR experiments. JGR was involved in sample preparation and clinical data management. EJH was involved in clinical data management. DMZ, LS, EA, JMS and EH were involved in family ascertainment, with LS, EA, JMS and EH supervising training and recruitment oversight. CB proposed and analyzed experiments and critically revised the manuscript. JDB was involved in the design and analysis of the study, and edited the manuscript.

## Pre-publication history

The pre-publication history for this paper can be accessed here:



## Supplementary Material

Additional File 1 **Chromosome position of genes scanned using MLPA probe with the associated genetic syndromes**. Data were adapted from documentation for SALSA MLPA KIT P064 MR1, SALSA MLPA KIT P096 MR2 and SALSA MLPA KIT P106 MRX (MRC-Holland, Amsterdam, Netherlands) and OMIM (NCBI). Click here for file

Additional File 2 **ADI-R and clinical features of individuals with duplications in 15q11 and 22q11.** Click here for file

Additional File 3 **ADI-R and sleep disorder features of individuals with duplications in PAR1/*ASMT *gene.** Click here for file
